# Association of C-Reactive Protein With Bacterial and Respiratory Syncytial Virus–Associated Pneumonia Among Children Aged <5 Years in the PERCH Study

**DOI:** 10.1093/cid/cix150

**Published:** 2017-05-29

**Authors:** Melissa M. Higdon, Tham Le, Katherine L. O’Brien, David R. Murdoch, Christine Prosperi, Henry C. Baggett, W. Abdullah Brooks, Daniel R. Feikin, Laura L. Hammitt, Stephen R. C. Howie, Karen L. Kotloff, Orin S. Levine, J. Anthony G. Scott, Donald M. Thea, Juliet O. Awori, Vicky L. Baillie, Stephanie Cascio, Somchai Chuananon, Andrea N. DeLuca, Amanda J. Driscoll, Bernard E. Ebruke, Hubert P. Endtz, Anek Kaewpan, Geoff Kahn, Angela Karani, Ruth A. Karron, David P. Moore, Daniel E. Park, Mohammed Ziaur Rahman, Rasheed Salaudeen, Phil Seidenberg, Somwe Wa Somwe, Mamadou Sylla, Milagritos D. Tapia, Scott L. Zeger, Maria Deloria Knoll, Shabir A. Madhi, for the PERCH Study Group, Katherine L. O’Brien, Orin S. Levine, Maria Deloria Knoll, Daniel R. Feikin, Andrea N. DeLuca, Amanda J. Driscoll, Nicholas Fancourt, Wei Fu, Laura L. Hammitt, Melissa M. Higdon, E. Wangeci Kagucia, Ruth A. Karron, Mengying Li, Daniel E. Park, Christine Prosperi, Zhenke Wu, Scott L. Zeger, Nora L. Watson, Jane Crawley, David R. Murdoch, W. Abdullah Brooks, Hubert P. Endtz, Khalequ Zaman, Doli Goswami, Lokman Hossain, Yasmin Jahan, Hasan Ashraf, Stephen R. C. Howie, Bernard E. Ebruke, Martin Antonio, Jessica McLellan, Eunice Machuka, Arifin Shamsul, Syed M.A. Zaman, Grant Mackenzie, J. Anthony G. Scott, Juliet O. Awori, Susan C. Morpeth, Alice Kamau, Sidi Kazungu, Micah Silaba Ominde, Karen L. Kotloff, Milagritos D. Tapia, Samba O. Sow, Mamadou Sylla, Boubou Tamboura, Uma Onwuchekwa, Nana Kourouma, Aliou Toure, Shabir A. Madhi, David P. Moore, Peter V. Adrian, Vicky L. Baillie, Locadiah Kuwanda, Azwifarwi Mudau, Michelle J. Groome, Nasreen Mahomed, Henry C. Baggett, Somsak Thamthitiwat, Susan A. Maloney, Charatdao Bunthi, Julia Rhodes, Pongpun Sawatwong, Pasakorn Akarasewi, Donald M. Thea, Lawrence Mwananyanda, James Chipeta, Phil Seidenberg, James Mwansa, Somwe Wa Somwe, Geoffrey Kwenda, Trevor P. Anderson, Joanne Mitchell

**Affiliations:** 1 Department of International Health, International Vaccine Access Center, Johns Hopkins Bloomberg School of Public Health, and; 2 Department of Pharmaceutical Health Services Research, University of Maryland,Baltimore;; 3 Department of Pathology, University of Otago, and; 4 Microbiology Unit, Canterbury Health Laboratories, Christchurch, New Zealand;; 5 Global Disease Detection Center, Thailand Ministry of Public Health–US Centers for Disease Control and Prevention Collaboration, Nonthaburi;; 6 Division of Global Health Protection, Center for Global Health, Centers for Disease Control and Prevention, Atlanta, Georgia;; 7 International Centre for Diarrhoeal Disease Research, Bangladesh (icddr,b), Dhaka and Matlab;; 8 Department of International Health, Johns Hopkins Bloomberg School of Public Health, Baltimore, Maryland;; 9 Division of Viral Diseases, National Center for Immunization and Respiratory Diseases, Centers for Disease Control and Prevention, Atlanta, Georgia;; 10 Kenya Medical Research Institute–Wellcome Trust Research Programme, Kilifi;; 11 Medical Research Council Unit, Basse, The Gambia;; 12 Department of Paediatrics, University of Auckland, and; 13 Centre for International Health, University of Otago, Dunedin, New Zealand;; 14 Division of Infectious Disease and Tropical Pediatrics, Department of Pediatrics, Center for Vaccine Development, Institute of Global Health, University of Maryland School of Medicine, Baltimore;; 15 Bill & Melinda Gates Foundation, Seattle, Washington;; 16 Department of Infectious Disease Epidemiology, London School of Hygiene & Tropical Medicine, United Kingdom;; 17 Center for Global Health and Development, Boston University School of Public Health, Massachusetts;; 18 Medical Research Council, Respiratory and Meningeal Pathogens Research Unit, and; 19 Department of Science and Technology/National Research Foundation, Vaccine Preventable Diseases Unit, University of the Witwatersrand, Johannesburg, South Africa;; 20 Nakhon Phanom Provincial Hospital, Thailand;; 21 Department of Epidemiology, Johns Hopkins Bloomberg School of Public Health, Baltimore, Maryland;; 22 Department of Clinical Microbiology and Infectious Diseases, Erasmus Medical Center, Rotterdam, The Netherlands;; 23 Fondation Mérieux, Lyon, France; Departments of; 24 Mental Health and; 25 International Health, Center for Immunization Research, Johns Hopkins Bloomberg School of Public Health, Baltimore, Maryland;; 26 Department of Paediatrics and Child Health, Chris Hani Baragwanath Academic Hospital and University of the Witwatersrand, Johannesburg, South Africa;; 27 Milken Institute School of Public Health, Department of Epidemiology and Biostatistics, George Washington University, District of Columbia;; 28 International Centre for Diarrhoeal Disease Research, Bangladesh (icddr,b), Dhaka;; 29 Medical Microbiology Department, Lagos University Teaching Hospital, Nigeria;; 30 Department of Emergency Medicine, University of New Mexico, Albuquerque;; 31 Department of Paediatrics and Child Health, School of Medicine, University of Zambia, Lusaka;; 32 Centre pour le Développement des Vaccins (CVD-Mali), Bamako; and; 33 Department of Biostatistics, Johns Hopkins Bloomberg School of Public Health, Baltimore, Maryland; 34 Johns Hopkins Bloomberg School of Public Health, Baltimore, Maryland; 35 Bill & Melinda Gates Foundation, Seattle, Washington; 36 Centers for Disease Control and Prevention [CDC], Atlanta, Georgia; 37 Emmes Corporation, Rockville, Maryland; 38 Nuffield Department of Clinical Medicine, University of Oxford, United Kingdom; 39 University of Otago, Christchurch, New Zealand; 40 icddr,b, Dhaka and Matlab, Bangladesh; 41 Medical Research Council, Basse, The Gambia; 42 KEMRI–Wellcome Trust Research Programme, Kilifi, Kenya; 43 Division of Infectious Disease and Tropical Pediatrics, Department of Pediatrics, Center for Vaccine Development, Institute of Global Health, University of Maryland School of Medicine, Baltimore, Maryland and Centre pour le Développement des Vaccins (CVD-Mali), Bamako, Mali; 44 Respiratory and Meningeal Pathogens Research Unit, University of the Witwatersrand, Johannesburg, South Africa; 45 Thailand Ministry of Public Health–US CDC Collaboration, Nonthaburi, Thailand; 46 Boston University School of Public Health, Boston, Massachusetts and University Teaching Hospital, Lusaka, Zambia; 47 Canterbury Health Laboratory, Christchurch, New Zealand

**Keywords:** C-reactive protein, bacteria, RSV, biomarker, pneumonia.

## Abstract

**Background.:**

Lack of a gold standard for identifying bacterial and viral etiologies of pneumonia has limited evaluation of C-reactive protein (CRP) for identifying bacterial pneumonia. We evaluated the sensitivity and specificity of CRP for identifying bacterial vs respiratory syncytial virus (RSV) pneumonia in the Pneumonia Etiology Research for Child Health (PERCH) multicenter case-control study.

**Methods.:**

We measured serum CRP levels in cases with World Health Organization–defined severe or very severe pneumonia and a subset of community controls. We evaluated the sensitivity and specificity of elevated CRP for “confirmed” bacterial pneumonia (positive blood culture or positive lung aspirate or pleural fluid culture or polymerase chain reaction [PCR]) compared to “RSV pneumonia” (nasopharyngeal/oropharyngeal or induced sputum PCR-positive without confirmed/suspected bacterial pneumonia). Receiver operating characteristic (ROC) curves were constructed to assess the performance of elevated CRP in distinguishing these cases.

**Results.:**

Among 601 human immunodeficiency virus (HIV)–negative tested controls, 3% had CRP ≥40 mg/L. Among 119 HIV-negative cases with confirmed bacterial pneumonia, 77% had CRP ≥40 mg/L compared with 17% of 556 RSV pneumonia cases. The ROC analysis produced an area under the curve of 0.87, indicating very good discrimination; a cut-point of 37.1 mg/L best discriminated confirmed bacterial pneumonia (sensitivity 77%) from RSV pneumonia (specificity 82%). CRP ≥100 mg/L substantially improved specificity over CRP ≥40 mg/L, though at a loss to sensitivity.

**Conclusions.:**

Elevated CRP was positively associated with confirmed bacterial pneumonia and negatively associated with RSV pneumonia in PERCH. CRP may be useful for distinguishing bacterial from RSV-associated pneumonia, although its role in discriminating against other respiratory viral-associated pneumonia needs further study.

C-reactive protein (CRP) is an acute phase plasma protein synthesized by hepatocytes and adipocytes in response to inflammatory cytokines and is an indicator of acute inflammation [1]. First identified in sera from pneumonia patients in 1930 by its ability to precipitate the C-polysaccharide of *Streptococcus pneumoniae* [2], CRP has since been associated with bacterial infections generally [3] and with noninfectious causes of inflammation [1, 4]. These associations have led to the use of CRP for discriminating between bacterial and nonbacterial pneumonia.

Several studies have found higher CRP levels in bacterial than viral pneumonia [5–16], whereas others have not [17–19]. Even in those detecting a difference, overlapping CRP distributions indicate imperfect specificity for bacterial pneumonia. The variation in reported utility of CRP for distinguishing etiologic class in pneumonia likely results from small sample sizes, lack of specific tests for accurately categorizing bacterial and viral pneumonia, and differences across studies in case groups, severity of disease, and comparison groups.

The Pneumonia Etiology Research for Child Health (PERCH) study provides an opportunity to examine the association between CRP and etiology of pneumonia in a number of children in several countries [20]. We describe the distribution of CRP among PERCH cases and a subset of community controls and examine factors associated with elevated CRP among both groups. We also evaluate the sensitivity and specificity of elevated CRP for bacterial pneumonia in comparison to pneumonia likely caused by respiratory syncytial virus (RSV), the most common respiratory virus associated with childhood pneumonia [21].

## METHODS

PERCH evaluated etiologic agents causing severe and very severe pneumonia among children <5 years of age in 9 sites across 7 countries: Dhaka and Matlab, Bangladesh; Basse, The Gambia; Kilifi, Kenya; Bamako, Mali; Soweto, South Africa; Nakhon Phanom and Sa Kaeo, Thailand; and Lusaka, Zambia [20].

Identification and selection of cases and controls have been described previously [22]. In brief, cases were hospitalized children aged 1–59 months with World Health Organization (WHO)–defined severe or very severe pneumonia [23]. Controls were randomly selected children from the community without severe or very severe pneumonia and frequency matched by age and month of enrollment to cases. In South Africa and Zambia where the human immunodeficiency virus (HIV) prevalence was high, controls were also frequency matched on HIV.

Blood, nasopharyngeal/oropharyngeal (NP/OP) swabs, and induced sputum were collected from PERCH cases at enrollment. Pleural fluid was collected from cases when clinically indicated. Lung aspirates were collected among a subset of cases meeting eligibility criteria [24] at the Bangladesh, The Gambia, Mali, and South Africa sites. Blood and NP/OP swabs were collected from PERCH controls.

Pathogen-specific testing methods by body fluid have also been described elsewhere [25]. In brief, NP/OP, induced sputum, lung aspirates, and pleural fluid were tested by quantitative real-time polymerase chain reaction (PCR) using the Fast Track Diagnostics Respiratory Pathogens 33 test (FTD Resp-33) (Fast-track Diagnostics, Sliema, Malta) for select viruses and bacteria. Lung aspirates and pleural fluid were also tested by Gram stain and bacterial culture. Whole blood among cases and controls was tested by real-time PCR for pneumococcus only; blood cultures were performed on cases using standardized automated systems.

CRP levels were measured in all PERCH cases from whom serum specimens were collected. To assess specificity for bacterial pneumonia, we evaluated elevated CRP among those most likely to have viral pneumonia, cases with RSV pneumonia (defined below). We also assessed CRP specificity by testing sera from a subset of community controls at each site, children who by definition did not have severe or very severe pneumonia, whether bacterial or otherwise. The subset of controls tested for CRP was enriched with children potentially more likely to have elevated CRP. This was achieved by oversampling from those who were positive for pneumococcus by whole-blood PCR, had a respiratory tract illness (defined below), had a total NP/OP PCR pathogen load (across all pathogens tested for) in the top 25% of controls at each site, or who were HIV-infected. Serum samples from South Africa were tested locally using CRP Gen3 Immunoturbidometric assay (Roche Diagnostics, Milan, Italy). Serum specimens from the other sites were tested for CRP at the PERCH reference laboratory in Christchurch, New Zealand, using CRP VARIO Immunoturbidometric assay (Roche Diagnostic, Milan, Italy).

### Definitions

Respiratory tract illness (RTI) in controls was defined as having cough or runny nose. RTI was also defined as having (1) at least 1 of ear discharge, wheezing, or difficulty breathing *and* (2) either a measured temperature of ≥38.0°C within the previous 48 hours or a history of sore throat. Chest radiograph positive (CXR+) was defined as chest radiograph performed up to 72 hours after presentation at study sites with evidence of alveolar consolidation (CXR-AC) or any other infiltrate (CXR-OI) by the WHO interpretation criteria [26, 27]. Confirmed bacterial pneumonia was defined as any noncontaminant bacterial pathogen detected by culture of blood; by PCR or culture of lung aspirate; or by PCR, culture, or pneumococcal antigen detection (BinaxNOW) of pleural fluid. Confirmed viral pneumonia was defined as any virus detected from lung aspirate or pleural fluid by PCR. Suspected bacterial pneumonia cases were cases who met all the following criteria: RSV-negative by NP/OP and induced sputum PCR; absence of confirmed bacterial and confirmed viral pneumonia; and NP/OP PCR *Streptococcus pneumoniae* (Spn) density >10^6.9^ copies/mL or whole-blood PCR Spn density >10^2.2^ copies/mL or NP/OP PCR *Haemophilus influenzae* (Hinf) density >10^5.9^ copies/mL (these PERCH thresholds that distinguished bacterial pneumonia cases due to Spn and Hinf from controls are described elsewhere in this supplement [28–30]). Respiratory syncytial virus positivity (RSV+) was defined as detection of RSV by PCR from NP/OP or induced sputum. For the purpose of this analysis, RSV pneumonia was defined as RSV+ cases without any of the following: confirmed bacterial pneumonia; high-density Spn (NP/OP PCR Spn density >10^6.9^ copies/mL or whole-blood PCR Spn density >10^2.2^ copies/mL); and high-density Hinf (NP/OP PCR Hinf density >10^5.9^ copies/mL).

### Statistical Analysis

Analyses were restricted to HIV-negative cases and controls unless otherwise noted. We evaluated the association of demographic and clinical factors with CRP ≥40 mg/L among CXR+ cases and among controls using logistic regression to adjust for age and site, with CRP ≥40 mg/L as the outcome. We also compared these characteristics among controls tested vs not tested for CRP. Because the subset of controls selected for CRP testing intentionally targeted elevated CRP, the proportion with elevated CRP is not representative of the prevalence in children in the community; therefore, we did not compare the distribution of CRP results between cases and controls.

Because there is no gold standard for determining viral etiology and because RSV was the only virus strongly associated with case-control status (odds ratio >7.0 at every site), we limited analyses of viral pneumonia to just RSV-associated pneumonia cases. We calculated the proportion of children with elevated CRP among subgroups with increasing likelihood of bacterial pneumonia (and decreasing likelihood of RSV pneumonia): CXR-normal cases, CXR-OI cases (without CXR-AC), and CXR-AC cases (with or without CXR-OI). We assessed the performance of CRP in distinguishing confirmed bacterial pneumonia from RSV pneumonia using receiver operating characteristic (ROC) analysis with the area under the curve (AUC) statistic [31]; the Youden index was used to determine the best differentiating cut-point [32]. To guard against bias in the estimates of sensitivity due to small numbers of confirmed cases, the Youden index was calculated using leave-one-out cross-validation where applicable [33].

Using Spn as an example, we explored whether additionally requiring elevated CRP could improve the specificity of pathogen-specific measures of high pathogen load (NP/OP Spn PCR density >10^6.9^ copies/mL; whole-blood Spn PCR density >10^2.2^ copies/mL) to identify Spn pneumonia. We compared the joint positivity of elevated CRP and high pathogen load among cases with confirmed Spn pneumonia to RSV+ cases that did not have a confirmed bacterial infection but may have had high-density Spn or Hinf. We also compared joint positivity among cases to the subset of controls that were selected for CRP testing.

Statistical analyses were performed using SAS software version 9.4 (SAS Institute, Cary, North Carolina). All *P* values provided were obtained from logistic regression analyses adjusting for age and site unless otherwise noted.

### Ethical Considerations

The PERCH study protocol was approved by the institutional review board or ethical review committee at each of the study site institutions and at the Johns Hopkins Bloomberg School of Public Health. Parents or guardians of all participants provided written informed consent.

## RESULTS

Among 3981 HIV-negative cases, 3357 (84%) had CRP measured. CRP ≥40 mg/L was observed in 28% of all HIV-negative cases and was more frequent among CXR+ cases (35%) than among CXR-normal cases (20%, *P* < .001; Table 1). CRP ≥100 mg/L was found in 11% of all HIV-negative cases and again was more common among CXR+ cases (15%) than CXR-normal cases (6%, *P* < .001; data not shown).

**Table 1.** Distribution of C-Reactive Protein Among Severe and Very Severe Pneumonia Cases—Pneumonia Etiology Research for Child Health (PERCH) Study

CRP Level, mg/L	All Cases	CXR Status				*P* Value^d^, All CXR+ vs CXR-Normal
		CXR-AC^a^	CXR-OI^b^	All CXR+^c^	CXR-Normal	
HIV-negative	(n = 3357)	(n = 729)	(n = 769)	(n = 1508)	(n = 1339)	
<10	1422 (42.4)	204 (27.6)	326 (42.4)	530 (35.2)	640 (47.8)	<.001
10 to <40	1010 (30.1)	199 (26.9)	253 (32.9)	452 (30.0)	435 (32.5)	
≥40	925 (27.6)	336 (45.5)	190 (24.7)	526 (34.9)	264 (19.7)	
HIV-positive	(n = 240)	(n = 120)	(n = 39)	(n = 159)	(n = 24)	
<10	83 (34.6)	33 (27.5)	13 (33.3)	46 (28.9)	10 (41.7)	.98
10 to <40	59 (24.6)	27 (22.5)	14 (35.9)	41 (25.8)	4 (16.7)	
≥40	98 (40.8)	60 (50.0)	12 (30.8)	72 (45.3)	10 (41.7)	

Data are presented as No. (%) unless otherwise indicated.

Abbreviations: AC, alveolar consolidation; CRP, C-reactive protein; CXR, chest radiograph; HIV, human immunodeficiency virus; OI, other infiltrate.

^a^CXR-AC: radiographic evidence of alveolar consolidation with or without any other infiltrate.

^b^CXR-OI: radiographic evidence of any other infiltrate without evidence of alveolar consolidation.

^c^All CXR+: radiographic evidence of alveolar consolidation, any other infiltrate, or both (includes both CXR-AC and CXR-OI cases).

^d^ *P* value comparing CRP <40 vs CRP ≥40 mg/L, adjusted for age and site.

### Factors Associated With Elevated CRP

CRP ≥40 mg/L was associated with HIV status: 45% of 159 HIV-positive CXR+ cases with available CRP results had CRP ≥40 mg/L compared with 35% of 1508 HIV-negative CXR+cases (*P* = .009). Among HIV-negative CXR+ cases, the proportion with CRP ≥40 mg/L was higher at the African sites (range, 31% in South Africa to 49% in The Gambia) than at Asian sites (13% in Bangladesh and 24% in Thailand). Additionally, among HIV-negative CXR+ cases, CRP ≥40 mg/L was more common among older children and those with very severe pneumonia, fever, or absence of wheeze (all *P* ≤ .001, adjusted for site and age; Supplementary Table 1). Among HIV-positive CXR+ cases, CRP ≥40 mg/L was similarly more common among older children and those with very severe pneumonia (*P* = .01 and *P* = .03, respectively, adjusted for site and age; data not shown).

### CRP Results Among Targeted Community Controls

By design, the 601 HIV-negative controls tested for CRP were more likely to have RTI (43%) and be PCR-positive for pneumococcus in whole blood (36%) than controls not tested (21% and 1%, respectively); 69% of tested controls met at least 1 of these conditions compared with 23% of controls not tested for CRP (Supplementary Table 2). Of tested controls, 12% (95% confidence interval [CI], 9%–15%) had CRP ≥10 mg/L and 3% (95% CI, 2%–4%) had CRP ≥40 mg/L. Of several factors examined, only pneumococcal PCR positivity in whole blood was associated with CRP ≥40 mg/L after adjusting for site and age. The proportion of tested HIV-negative controls with CRP ≥40 mg/L varied by site, ranging from 0% in Zambia and South Africa to 7% in The Gambia. CRP ≥40 mg/L was also more frequent among controls who had at least 1 of RTI, NP/OP Spn density >10^6.9^ copies/mL, or whole-blood PCR positivity for pneumococcus (3% vs 1% among controls with none of these characteristics).

Of 221 HIV-positive controls enrolled at the South African and Zambian sites, 81 (37%) were tested for CRP. CRP ≥40 mg/L was more common among the HIV-positive (11%; 95% CI, 4%–18%) than the HIV-negative (3%) controls selected for testing across all PERCH sites as was CRP ≥10 mg/L (30% vs 12%, data not shown). No factors were found to be associated with CRP ≥40 mg/L among the HIV-positive controls, but sample size was small (data not shown).

### Association of Elevated CRP With Bacterial Versus RSV Pneumonia

Of 842 HIV-negative RSV+ cases, 286 (34%) were excluded from the RSV pneumonia case group because they had a confirmed bacterial infection (n = 9), high-density Spn in the NP/OP (n = 111) or whole blood (n = 22), or high-density Hinf in the NP/OP (n = 199).

Among 119 HIV-negative cases with confirmed bacterial pneumonia, 77% had CRP ≥40 mg/L compared with 17% of 556 cases with RSV pneumonia (*P* < .001). Of the 286 excluded RSV+ cases, 85 (30%) had CRP ≥40 mg/L.

Among HIV-positive cases, differences were less extreme but trends were similar, though small numbers limit interpretation: 69% of 26 cases with confirmed bacterial pneumonia had CRP ≥40 mg/L compared with 45% of 11 cases with RSV pneumonia (data not shown; *P* = .41).

**Figure 1.**
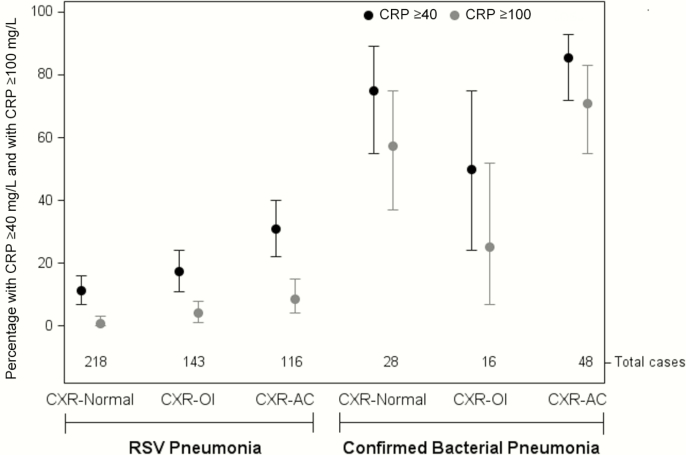
Percentage of cases with elevated (≥40 mg/L) and high (≥100 mg/L) C-reactive protein by (approximate) increasing likelihood of bacterial etiology. Confirmed bacterial pneumonia: bacterial pathogen identified by blood culture, by culture or polymerase chain reaction (PCR) of lung aspirate or pleural fluid, or *Streptococcus pneumoniae* identified by BinaxNOW assay of pleural fluid. Respiratory syncytial virus (RSV) pneumonia: RSV identified by nasopharyngeal/oropharyngeal (NP/OP) PCR or induced sputum PCR excluding (1) confirmed bacterial cases and (2) cases with whole-blood pneumococcal density >10^2.2^ copies/mL, or NP/OP pneumococcal PCR density >10^6.9^ copies/mL, or *Haemophilus influenzae* NP/OP PCR density >10^5.9^ copies/mL. Vertical bars: 95% confidence intervals. Abbreviations: CXR+, cases with radiographic evidence of alveolar consolidation, any other infiltrate, or both; CXR-AC, cases with radiographic evidence of alveolar consolidation (with or without any other infiltrate); CXR-OI, cases with radiographic evidence of any other infiltrate only.

An abnormal chest radiograph was associated with CRP ≥40 mg/L among HIV-negative cases with RSV pneumonia (24% of CXR+ vs 12% of CXR-normal cases, *P* < .001) but not cases with confirmed bacterial pneumonia (77% vs 75%; Supplementary Table 4). However, among CXR+ cases in both groups, the percentage with elevated CRP was higher among cases with CXR-AC than cases with CXR-OI: 85% vs 50% (*P* = .004) among confirmed bacterial pneumonia cases and 31% vs 18% (*P* = .01) among RSV pneumonia cases (Figure 1, Supplementary Table 4). High CRP (≥100 mg/L) was very common among the CXR-AC cases with confirmed bacterial pneumonia (71%) and uncommon (4%) among RSV pneumonia cases (Figure 1).

Among the confirmed bacterial pneumonia cases, those confirmed for either Spn or Hinf had higher CRP (84% CRP ≥40 mg/L and 74% CRP ≥100 mg/L) than cases confirmed for other bacteria (69% CRP ≥40 mg/L, *P* = .18 and 45% CRP ≥100 mg/L, *P* = .02; Supplementary Table 5).

**Figure 2.**
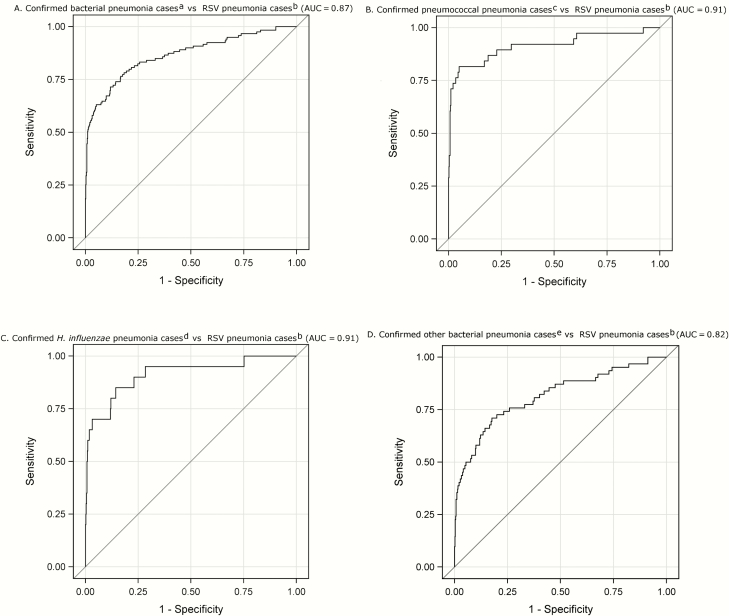
*A–D*, Receiver operating characteristic curves and area under the curve (AUC) for C-reactive protein in differentiating confirmed bacterial pneumonia from probable respiratory syncytial virus (RSV) pneumonia among human immunodeficiency virus–negative Pneumonia Etiology Research for Child Health (PERCH) cases. ^a^Cases with any noncontaminant bacteria identified by blood culture, by culture or polymerase chain reaction (PCR) of lung aspirate or pleural fluid, or with *Streptococcus pneumoniae* identified by BinaxNOW assay of pleural fluid. ^b^RSV identified by nasopharyngeal/oropharyngeal (NP/OP) PCR or induced sputum PCR, excluding high-density bacterial cases (*Streptococcus pneumoniae* cases with whole-blood PCR density >10^2.2^ copies/mL, or NP/OP density >10^6.9^ copies/mL, and *Haemophilus influenzae* cases with NP/OP density >10^5.9^ copies/mL) and confirmed bacterial cases. ^c^Cases with *S. pneumoniae* identified by blood culture, by lung aspirate culture or PCR, or by pleural fluid culture or PCR or BinaxNOW. ^d^Cases with *H. influenzae* identified by blood culture, by lung aspirate culture or PCR, or by pleural fluid culture or PCR. ^e^Cases with a bacterial pathogen identified by blood culture, by lung aspirate culture or PCR, or by pleural fluid culture or PCR (excluding confirmed *S. pneumoniae* and *H. influenzae* cases).

ROC analyses showed that CRP had good accuracy in distinguishing cases with confirmed bacterial infection from RSV pneumonia cases (AUC = 0.87, Figure 2); the CRP cut-point that produced optimal differentiation was 37.1 mg/L with a corresponding sensitivity of 77% (95% CI, 69%–84%) and specificity of 82% (95% CI, 78%–85%). When trying to distinguish confirmed Spn cases from RSV pneumonia, the AUC increased to 0.91; the optimal cut-point was 88.9 mg/L, resulting in a sensitivity and specificity of 79% (95% CI, 64%–89%) and 95% (95% CI, 93%–97%), respectively. The AUC for distinguishing confirmed Hinf cases from RSV pneumonia cases was also 0.91; the optimal cut-point of 52.3 mg/L produced a sensitivity and specificity of 80% (95% CI, 58%–92%) and 86% (95% CI, 83%–89%), respectively.

### Value of CRP to Increase Specificity of Other Etiology Laboratory Measurements

We assessed how combining elevated CRP with Spn density measures improved the specificity over Spn density measures alone in differentiating confirmed pneumococcal cases from: (1) RSV+ cases without confirmed bacterial coinfection and (2) community controls tested for CRP. The percentage of RSV+ cases and tested community controls with both CRP ≥40 mg/L and high density Spn in the NP/OP was 4% and 1%, respectively, compared with 13% and 12%, respectively, with high NP/OP density alone. This gain in specificity came without substantial loss in sensitivity, declining from 58% of confirmed Spn cases with high NP/OP density to 47% who also had CRP ≥40 mg/L (Table 2). Less than 1% of RSV+ cases and 0% of tested controls had high Spn NP/OP density and CRP ≥100 mg/L compared with 42% of confirmed Spn cases.

**Table 2.** C-Reactive Protein Combined With Pneumococcal Nasopharyngeal/Oropharyngeal and Whole-Blood Polymerase Chain Reaction Density Measures for Distinguishing Confirmed Pneumococcal Pneumonia From Respiratory Syncytial Virus–Positive Cases, Controls Targeted for CRP Testing, and Confirmed Other Bacterial Cases

Density Measure	Confirmed Spn Cases^a^	RSV+ Cases^b^	Controls Targeted for CRP Testing^c^	Confirmed Non- Spn Bacterial Cases^d^
NP/OP PCR Spn density	(n = 36)	(n = 858)	(n = 597)	(n = 79)
NP/OP PCR density > 10^6.9^ copies/mL^e^ alone	21 (58.3)	109 (12.7)	73 (12.2)	20 (25.3)
+ CRP ≥40 mg/L	17 (47.2)	33 (3.9)	3 (0.5)	17 (21.5)
+ CRP ≥100 mg/L	15 (41.7)	8 (0.9)	0 (0.0)	14 (17.7)
Whole-blood PCR Spn density	(n = 35)	(n = 847)	(n = 597)	(n = 78)
Density >10^2.2^ copies/mL^e^ alone	18 (51.4)	19 (2.2)	119 (19.9)	4 (5.1)
+ CRP ≥40 mg/L	17 (48.6)	3 (0.4)	5 (0.8)	3 (3.9)
+ CRP ≥100 mg/L	16 (45.7)	1 (0.1)	1 (0.2)	1 (1.3)
NP/OP PCR Spn density *or* whole-blood PCR Spn density	(n = 34)	(n = 835)	(n = 593)	(n = 77)
(NP/OP density >10^6.9^ copies/mL *or* whole-blood density >10^2.2^ copies/mL)^e^ alone	27 (79.4)	126 (15.1)	179 (30.2)	23 (29.9)
+ CRP ≥40 mg/L	23 (67.7)	35 (4.2)	8 (1.4)	19 (24.7)
+ CRP ≥100 mg/L	21 (61.8)	9 (1.1)	1 (0.2)	15 (19.5)
NP/OP PCR Spn density *and* whole-blood PCR Spn density	(n = 37)	(n = 870)	(n = 601)	(n = 80)
(NP/OP density >10^6.9^ copies/mL *and* whole-blood density >10^2.2^ copies/mL)^e^ alone	12 (32.4)	2 (0.2)	13 (2.2)	1 (1.3)
+ CRP ≥40 mg/L	11 (29.7)	1 (0.1)	0 (0.0)	1 (1.3)
+ CRP ≥100 mg/L	10 (27.0)	0 (0.0)	0 (0.0)	0 (0.0)

Data are presented as No. (%) positive unless otherwise indicated.

Abbreviations: CRP, C-reactive protein; NP/OP, nasopharyngeal/oropharyngeal; PCR, polymerase chain reaction; RSV, respiratory syncytial virus; Spn, *Streptococcus pneumoniae*.

^a^Spn detected by blood culture, by lung aspirate culture or PCR, or by pleural fluid culture or PCR or BinaxNOW assay.

^b^RSV detected by NP/OP PCR or induced sputum PCR, excluding confirmed Spn cases and confirmed other bacterial cases.

^c^Controls selected for CRP testing were children more likely to have elevated CRP levels (a higher proportion had respiratory symptoms, were whole-blood Spn PCR positive, and/or had high total NP/OP pathogen load compared to controls not tested).

^d^Any non-Spn bacterial pathogen detected by blood culture, by lung aspirate culture or PCR, or by pleural fluid culture or PCR.

^e^Cut-points obtained from receiver operating characteristic analyses that maximized Youden index in distinguishing confirmed Spn cases from community controls.

Because the optimal whole-blood Spn PCR density cut-point identified in ROC analyses had high specificity as a single measure (only 2% of RSV+ cases had high density Spn in whole blood), also requiring CRP ≥40 mg/L increased specificity only minimally (0.4% positive for both measurements). However, the gain in specificity was larger when compared to the tested community controls: 20% had high-density Spn in whole blood compared with 0.8% who also had CRP ≥40 mg/L_._

## DISCUSSION

This large study of hospitalized severe and very severe pneumonia from 7 countries in Africa and Asia showed that elevated CRP was positively associated with confirmed bacterial pneumonia and negatively associated with RSV pneumonia as defined for this analysis. The percentage of cases with CRP ≥40 mg/L varied by site, age, HIV status, CXR findings, severity, presence of fever, and wheeze. Among community controls targeted for CRP testing because of suspected potential for elevated CRP, the proportion with CRP ≥40 mg/L was low (3% among HIV-negative controls and 11% among HIV-positive controls) but specificity was not 100%.

We conducted this analysis to see if CRP could be a useful diagnostic to distinguish bacterial from viral pneumonia. But because there is no gold standard for diagnosing viral pneumonia, we limited our analysis of viruses solely to RSV since RSV was the only viral pathogen assessed that was both rarely observed in the NP/OP of controls and strongly associated with case status. That evidence suggested RSV was causally associated with the pneumonia episode in a large fraction of the RSV+ cases. However, severe and very severe pneumonia associated with RSV may not be representative of pneumonias associated with other viruses as viruses might differ in their propensity to cause bacterial super- or coinfections. In our analyses, we excluded RSV+ cases who may have had bacterial coinfection, an important and potentially substantial subgroup of cases with bacterial–viral coinfection. Other studies of children with respiratory infections that have observed increased CRP levels among cases with certain viruses detected have a similar problem of inability to rule out concurrent bacterial infections and inability to confirm viral etiology because of lack of gold standard tests [34–37]. Because our analyses assessed a case group that likely had true RSV pneumonia, we concluded that elevated CRP levels were more common in bacterial than RSV pneumonia. However, our conclusions cannot extend to other viruses as the negative association found in PERCH between RSV pneumonia and elevated CRP may not be true of all viruses.

Another challenge in interpreting elevated CRP was that the proportion with CRP ≥40 mg/L varied by bacterial pathogen; CRP was higher among cases confirmed for Spn and Hinf than cases confirmed for other bacteria. Although sample size was limited and the difference was not statistically significant, this may suggest that the CRP response could vary by bacterial pathogen. However, it is also possible that this indicates imperfect specificity of determining bacterial pneumonia, as the majority of the “confirmed” bacterial cases were diagnosed based on detection of bacteria in the blood and may not represent the infection in the lung.

If the distribution of CRP differs by bacteria, then the relative distribution of bacterial etiologies among the cases will affect the calculation of CRP cut-points for distinguishing bacterial from RSV pneumonia cases. Because the CRP cut-points calculated in this analysis were selected to maximize the sum of sensitivity and specificity for our data, they may not be representative of other settings and are presented for descriptive purposes only. However, they may have utility in determining or corroborating etiology results within the PERCH study.

In clinical practice, a test that has both high specificity and sensitivity to identify bacterial infection would be helpful to identify those children who would benefit from antibiotic therapy. The WHO definition of severe and very severe pneumonia was designed to be very sensitive (at the expense of specificity), to identify as many cases of bacterial etiology as possible in resource-poor settings for treatment with antibiotics. As a result, many cases meeting the definition do not have a bacterial infection. While CRP ≥40 mg/L may be a fairly specific marker to rule out most RSV pneumonias (83% of RSV pneumonia cases had CRP <40 mg/L), it had inadequate sensitivity as 23% of cases with confirmed bacterial severe or very severe pneumonia also had CRP <40 mg/L. Our findings confirm the current consensus in the literature, which is that while CRP is elevated in bacterial pneumonia, CRP alone is not sufficient for diagnosing bacterial pneumonia.

In addition to being able to distinguish between cases with bacterial vs RSV pneumonia, the informative value of CRP in etiology studies includes its ability to distinguish between bacterial pneumonia cases and controls without pneumonia. CRP levels among controls can be used to determine a reasonable minimal threshold to serve as a reliable biomarker for bacterial pneumonia. Reports of CRP levels in healthy children 1–59 months in the published literature are few and often have limited sample sizes (≤100 controls) or include older children [38–42]; thus, this analysis serves to anchor any conclusions on the practical application of the utility of CRP levels as a diagnostic tool for bacterial pneumonia in this age group. Though, because the controls selected for testing in our study deliberately targeted those thought to have a higher likelihood of elevated CRP, an analysis comparing controls to cases would have been biased by underestimating specificity for bacterial pneumonia. Even with the targeted sampling of controls for CRP testing, 88% of tested HIV-negative controls had CRP levels <10 mg/L. Nevertheless, the 3% found with CRP ≥40 mg/L demonstrates the lack of perfect specificity of this marker for identifying bacterial pneumonia. Of the 168 tested HIV-negative controls who did not have an RTI nor high-density Spn in NP/OP nor Spn detected in whole blood by PCR, 1 (0.6%) still had CRP ≥40 mg/L. This child, who had a CRP level of 81.3 mg/L, was 5 months of age from the Mali site with no signs of illness or malnutrition and no apparent factors for elevated CRP.

Combining CRP with pathogen-specific measurements increased their specificity for distinguishing bacterial from RSV etiology as we demonstrated for Spn without substantial loss to sensitivity. But CRP does not distinguish between bacterial etiologies even though CRP was somewhat higher among cases confirmed for Spn or Hinf than among cases confirmed for other bacteria.

For pneumonia, identifying cases with a confirmed etiology is possible in only a small subset because lung aspirates and pleural fluid specimens are obtained from very few cases and blood culture has low sensitivity, especially when blood culture volume is low or antibiotics are administered prior to specimen collection. Therefore, because few bacterial pneumonia cases have a confirmed etiology, the RSV pneumonia cases in our analysis could have included children with undetected bacterial coinfection. Misclassifications of this type would result in overestimating the proportion of RSV pneumonia cases with elevated CRP, and thus the specificity of CRP for detecting bacterial compared to viral pneumonia may be higher.

Our analyses showed that elevated CRP was positively associated with confirmed bacterial pneumonia, especially Spn and Hinf, and negatively associated with RSV pneumonia. However, the variation in the distribution of CRP among study sites for both cases and controls and by clinical factors such as HIV suggests that optimal cut-points for diagnostic utility may vary by setting or geographic location. While CRP had imperfect specificity for distinguishing bacterial from RSV pneumonia and therefore limited use as a diagnostic tool, the clear association of elevated CRP with bacterial pneumonia makes it potentially useful in epidemiologic studies on bacterial pneumonia, as cases with low CRP could be assumed to have lower probability of bacterial etiology than cases with high CRP. The role of CRP in discriminating between bacterial pneumonia and viral pneumonias other than RSV warrants further study.

## Supplementary Data

Supplementary materials are available at *Clinical Infectious Diseases* online. Consisting of data provided by the authors to benefit the reader, the posted materials are not copyedited and are the sole responsibility of the authors, so questions or comments should be addressed to the corresponding author.

## Supplementary Material

Supplementary_Materials

## Notes

***Author contributions.*** M. M. H. led the analysis, interpreted results, and drafted initial manuscript. T. L. and G. K. performed the analysis. K. L. O., D. R. M., C. P., M. D. K., and S. A. M. provided significant guidance on the development of the manuscript. K. L. O., D. R. M., H. C. B., W. A. B., D. R. F., L. L. H., S. R. C. H., K. L. K., O. S. L., J. A. G. S., D. M. T., M. D. K., S. A. M., and R. A. K. conceived and designed the study and supervised study conduct. M. M. H., C. P., J. O. A., V. L. B., S. Ch., A. N. D., A. J. D., B. E. E., H. E., A. Kae., A. Kar., D. P. M., D. E. P., M. R., R. S., P. S., S. W. S., M. S., and M. D. T. were involved in study conduct. S. C. assisted with the drafting of the manuscript. S. L. Z. provided statistical expertise and led the integrated etiology analysis. All authors reviewed and approved the manuscript. M. M. H. had full access to all the data in the study and had final responsibility for the decision to submit for publication.

***Acknowledgments.*** We acknowledge the significant contributions of the PERCH Study Group and all PERCH investigators. We offer our gratitude to the members of the Pneumonia Methods Working Group, PERCH Expert Group, and PERCH Chest Radiograph Reading Panel for their time and lending expertise to assist the PERCH Study Group (see Supplementary Materials for a full list of names). We offer sincere thanks to the patients and families who participated in this study.

***Financial support.*** PERCH was supported by the Bill & Melinda Gates Foundation (grant number 48968 to the International Vaccine Access Center, Department of International Health, Johns Hopkins Bloomberg School of Public Health). J. A. G. S. was supported by a clinical fellowship from the Wellcome Trust of Great Britain (award number 098532).

***Supplement sponsorship.*** This article appears as part of the supplement “Pneumonia Etiology Research for Child Health (PERCH): Foundational Basis for the Primary Etiology Results,” sponsored by a grant from the Bill & Melinda Gates Foundation to the PERCH study of Johns Hopkins Bloomberg School of Public Health, Baltimore, Maryland.

***Potential conflicts of interest.*** M. D. K. has received funding for consultancies from Merck, Pfizer, and Novartis, and grant funding from Merck. L. L. H. has received grant funding from Pfizer and GlaxoSmithKline. K. L. K. has received grant funding from Merck Sharp & Dohme. S. A. M. has received honoraria for advisory board membership from the Bill & Melinda Gates Foundation, Pfizer, Medimmune, and Novartis; has received institutional grants from GSK, Novartis, Pfizer, Minervax, and the Bill & Melinda Gates Foundation; and has served on speaker’s bureaus for Sanofi Pasteur and GSK. K. L. O. has received grant funding from Pfizer and GlaxoSmithKline and has served on technical advisory boards for Merck, Sanofi Pasteur, PATH, Affinivax, and ClearPath. All other authors report no potential conflicts. All authors have submitted the ICMJE Form for Disclosure of Potential Conflicts of Interest. Conflicts that the editors consider relevant to the content of the manuscript have been disclosed.
