# Supplementary material for: Association of C-Reactive Protein With Bacterial and Respiratory Syncytial Virus–Associated Pneumonia Among Children Aged <5 Years in the PERCH Study
Source: Clin Infect Dis. 2017 May 29;64(Suppl 3):S378–86. doi: 10.1093/cid/cix150 (PMC5447856; doi:10.1093/cid/cix150)
Supplement: Supplementary_Materials [file cix150_suppl_Supplementary_Materials.docx]

**Supplementary Table 1. Factors associated with elevated C-reactive protein (CRP) among HIV-negative, CXR-positive^a^ cases**

|  | **All**  **(N=1508)** | **CRP <10 mg/L**  **(N=530)** | **CRP 10-<40 mg/L**  **(N=452)** | **CRP ≥40 mg/L**  **(N=526)** | **<40 vs. ≥40 mg/L** | |
| --- | --- | --- | --- | --- | --- | --- |
|  | n (col%) | n (row%) | n (row%) | n (row%) | **p-value**^b^ | **adjusted p-value**^c^ |
| **Site** |  |  |  |  |  |  |
| Kenya | 217 (14.4) | 58 (26.7) | 81 (37.3) | 78 (35.9) | <.001 | <.001 |
| The Gambia | 176 (11.7) | 35 (19.9) | 55 (31.3) | 86 (48.9) |  |  |
| Mali | 207 (13.7) | 75 (36.2) | 43 (20.8) | 89 (43.0) |  |  |
| Zambia | 188 (12.5) | 57 (30.3) | 40 (21.3) | 91 (48.4) |  |  |
| South Africa | 427 (28.3) | 160 (37.5) | 133 (31.1) | 134 (31.4) |  |  |
| Thailand | 91 (6.0) | 35 (38.5) | 34 (37.4) | 22 (24.2) |  |  |
| Bangladesh | 202 (13.4) | 110 (54.5) | 66 (32.7) | 26 (12.9) |  |  |
| **Age** (months) |  |  |  |  |  |  |
| 1-5 | 590 (39.1) | 251 (42.5) | 171 (29.0) | 168 (28.5) | <.001 | <.001 |
| 6-11 | 359 (23.8) | 133 (37.0) | 100 (27.9) | 126 (35.1) |  |  |
| 12-23 | 374 (24.8) | 102 (27.3) | 121 (32.4) | 151 (40.4) |  |  |
| 24-59 | 185 (12.3) | 44 (23.8) | 60 (32.4) | 81 (43.8) |  |  |
| **Sex** |  |  |  |  |  |  |
| Male | 855 (56.7) | 292 (34.2) | 266 (31.1) | 297 (34.7) | .89 | .99 |
| Female | 653 (43.3) | 238 (36.4) | 186 (28.5) | 229 (35.1) |  |  |
| **Severity** |  |  |  |  |  |  |
| Severe | 1073 (71.2) | 387 (36.1) | 342 (31.9) | 344 (32.1) | <.001 | .001 |
| Very severe | 435 (28.8) | 143 (32.9) | 110 (25.3) | 182 (41.8) |  |  |
| **Malnutrition**^d^ |  |  |  |  |  |  |
| Normal (WAZ > -2 SDs) | 962 (64.0) | 342 (35.6) | 297 (30.9) | 323 (33.6) | .14 | .11 |
| Moderate/Severe (WAZ < -2 SDs) | 540 (36.0) | 184 (34.1) | 154 (28.5) | 202 (37.4) |  |  |
| **Prior antibiotic use**^e^ |  |  |  |  |  |  |
| No | 653 (53.8) | 201 (30.8) | 197 (30.2) | 255 (39.1) | .78 | .38 |
| Yes | 560 (46.2) | 183 (32.7) | 154 (27.5) | 223 (39.8) |  |  |
| **Observed fever** |  |  |  |  |  |  |
| No | 1027 (68.2) | 413 (40.2) | 324 (31.5) | 290 (28.2) | <.001 | <.001 |
| Yes | 478 (31.8) | 115 (24.1) | 127 (26.6) | 236 (49.4) |  |  |
| **Observed or reported fever** |  |  |  |  |  |  |
| No | 267 (17.7) | 149 (55.8) | 77 (28.8) | 41 (15.4) | <.001 | <.001 |
| Yes | 1240 (82.3) | 380 (30.6) | 375 (30.2) | 485 (39.1) |  |  |
| **Duration of illness (days)**^f^ |  |  |  |  |  |  |
| <3 | 441 (29.4) | 149 (33.8) | 151 (34.2) | 141 (32.0) | .25 | .34 |
| 3-5 | 719 (48.0) | 240 (33.4) | 225 (31.3) | 254 (35.3) |  |  |
| >5 | 338 (22.6) | 137 (40.5) | 74 (21.9) | 127 (37.6) |  |  |
| **Hypoxemia**^g^ |  |  |  |  |  |  |
| No | 838 (55.6) | 307 (36.6) | 247 (29.5) | 284 (33.9) | .34 | .37 |
| Yes | 668 (44.4) | 222 (33.2) | 204 (30.5) | 242 (36.2) |  |  |
| **Wheezing** |  |  |  |  |  |  |
| No | 1065 (70.7) | 327 (30.7) | 301 (28.3) | 437 (41.0) | <.001 | <.001 |
| Yes | 440 (29.3) | 201 (45.7) | 151 (34.3) | 88 (20.0) |  |  |
| **Died within 30 days**^h^ |  |  |  |  |  |  |
| No | 1245 (92.9) | 443 (35.6) | 389 (31.2) | 413 (33.2) | .25 | .88 |
| Yes | 95 (7.1) | 40 (42.1) | 18 (18.9) | 37 (38.9) |  |  |
| **Hospital death** |  |  |  |  |  |  |
| No | 1430 (94.9) | 498 (34.8) | 439 (30.7) | 493 (34.5) | .13 | .59 |
| Yes | 77 (5.1) | 32 (41.6) | 12 (15.6) | 33 (42.9) |  |  |
| **Hib and PCV vaccination^i^** |  |  |  |  |  |  |
| None | 250 (21.1) | 105 (42.0) | 77 (30.8) | 68 (27.2) | <.001 | .75 |
| ≥1 HiB dose & no PCV | 175 (14.7) | 74 (42.3) | 55 (31.4) | 46 (26.3) |  |  |
| ≥1 HiB & ≥1 PCV dose | 762 (64.2) | 231 (30.3) | 236 (31.0) | 295 (38.7) |  |  |
| ^a^ CXR-positive: evidence of alveolar consolidation, other infiltrate, or both on chest radiograph. | | | | | | |
| ^b^ Chi-square unadjusted. | | | | | | |
| ^c^ P-value obtained from multivariate logistic regression adjusting for site and age, comparing CRP<40 vs. CRP≥40 mg/l. | | | | | | |
| ^d^ Moderate or severe malnutrition (WHO wt-for-age z score < -2 SDs). | | | | | | |
| ^e^ Antibiotic pretreatment: administered antibiotics at the study facility prior to the collection of specimens (cases only), positive serum bioassay, received antibiotics at a referral facility. | | | | | | |
| ^f^ Number of days of cough, fever, runny nose, difficulty breathing, ear discharge, or wheeze prior to enrollment, whichever is longest. | | | | | | |
| ^g^ Hypoxemia: room air reading of O_2_ sat <90 at sites at elevation (Zambia and South Africa) and <92 at all other sites OR O_2_ requirement (if on O_2_ and room air reading not available) | | | | | | |
| ^h^ Any death within 30 days of enrollment among cases with known vital status at 30 days. | | | | | | |
| ^i^ Hib, H. influenzae type b vaccine; PCV, pneumococcal conjugate vaccine. | | | | | | |

| **Supplementary Table** **2. Characteristics of HIV-negative controls targeted vs. not targeted for C-reactive protein (CRP) testing and distribution of CRP among tested controls** | | | | | | | |
| --- | --- | --- | --- | --- | --- | --- | --- |
|  | **All controls** | | | **CRP distribution among controls targeted for CRP testing^a^** | | | |
|  | **Tested** | **Not tested** | **p-value^b^** | **< 10 mg/L** | **10-<40 mg/L** | **≥ 40 mg/L** | **p-value^c^** |
|  | n (col %) | n (col %) |  | n (row %) | n (row %) | n (row %) |  |
| **Overall** | n=601 | n=4501 |  | 530 (88.2) | 55 (9.2) | n=16 (2.7) |  |
| **Site** |  |  |  |  |  |  |  |
| Kenya | 50 (8.3) | 813 (18.1) | <.001 | 42 (84.0) | 6 (12.0) | 2 (4.0) | .06 |
| Gambia | 84 (14.0) | 570 (12.7) |  | 68 (81.0) | 10 (11.9) | 6 (7.1) |  |
| Mali | 94 (15.6) | 631 (14.0) |  | 79 (84.0) | 9 (9.6) | 6 (6.4) |  |
| Zambia | 40 (6.7) | 561 (12.5) |  | 33 (82.5) | 7 (17.5) | 0 (0.0) |  |
| South Africa | 151 (25.1) | 677 (15.0) |  | 140 (92.7) | 11 (7.3) | 0 (0.0) |  |
| Thailand | 89 (14.8) | 570 (12.7) |  | 82 (92.1) | 6 (6.7) | 1 (1.1) |  |
| Bangladesh | 93 (15.5) | 679 (15.1) |  | 86 (92.5) | 6 (6.5) | 1 (1.1) |  |
| **Age (months)** |  |  |  |  |  |  |  |
| 1-5 | 157 (26.1) | 1441 (32.0) | .02 | 141 (89.8) | 13 (8.3) | 3 (1.9) | .73 |
| 6-11 | 155 (25.8) | 1055 (23.4) |  | 134 (86.5) | 17 (11.0) | 4 (2.6) |  |
| 12-23 | 168 (28.0) | 1094 (24.3) |  | 146 (86.9) | 17 (10.1) | 5 (3.0) |  |
| 24-59 | 121 (20.1) | 911 (20.2) |  | 109 (90.1) | 8 (6.6) | 4 (3.3) |  |
| **Respiratory Tract Illness (RTI)^d^** |  |  |  |  |  |  |  |
| No | 342 (56.9) | 3554 (79.0) | <.001 | 307 (89.8) | 24 (7.0) | 11 (3.2) | .12 |
| Yes | 259 (43.1) | 947 (21.0) |  | 223 (86.1) | 31 (12.0) | 5 (1.9) |  |
| ***S. pneumoniae* (Spn)** |  |  |  |  |  |  |  |
| **NP/OP PCR** |  |  |  |  |  |  |  |
| Negative | 110 (18.4) | 1026 (23.4) | .006 | 102 (92.7) | 7 (6.4) | 1 (0.9) | .34 |
| Positive | 488 (81.6) | 3353 (76.6) |  | 425 (87.1) | 48 (9.8) | 15 (3.0) |  |
| **NP/OP PCR density** |  |  |  |  |  |  |  |
| ≤ 10^6.9^ copies/ml | 524 (87.6) | 4073 (93.0) | <.001 | 461 (88.0) | 51 (9.7) | 12 (2.3) | .16 |
| > 10^6.9^ copies/ml | 74 (12.4) | 306 (7.0) |  | 66 (89.2) | 4 (5.4) | 4 (5.4) |  |
| **Whole blood PCR** |  |  |  |  |  |  |  |
| Negative | 384 (64.3) | 4140 (99.0) | <.001 | 347 (90.4) | 33 (8.6) | 4 (1.0) | .005 |
| Positive | 213 (35.7) | 42 (1.0) |  | 180 (84.5) | 22 (10.3) | 11 (5.2) |  |
| **Whole blood PCR density** |  |  |  |  |  |  |  |
| ≤ 10^2.2^ copies/ml | 478 (80.1) | 4160 (99.5) | <.001 | 426 (89.1) | 42 (8.8) | 10 (2.1) | .44 |
| > 10^2.2^ copies/ml | 119 (19.9) | 22 (0.5) |  | 101 (84.9) | 13 (10.9) | 5 (4.2) |  |
| **RTI or whole blood Spn PCR+** |  |  |  |  |  |  |  |
| No | 187 (31.1) | 3267 (76.9) | <.001 | 172 (92.5) | 11 (5.9) | 3 (1.6) | .61 |
| Yes | 414 (68.9) | 984 (23.2) |  | 357 (86.2) | 44 (10.6) | 13 (3.1) |  |
| **RTI or whole blood Spn PCR+ or NP/OP Spn density >10^6.9^ copies/ml** |  |  |  |  |  |  |  |
| No | 168 (28.1) | 2969 (71.0) | <.001 | 157 (93.5) | 10 (6.0) | 1 (0.6) | .14 |
| Yes | 430 (71.9) | 1215 (29.0) |  | 370 (86.1) | 45 (10.5) | 15 (3.5) |  |
| **Malnutrition^e^** |  |  |  |  |  |  |  |
| Normal (WAZ > -2 SDs) | 523 (87.5) | 3947 (88.1) | .66 | 462 (88.3) | 47 (9.0) | 14 (2.7) | .72 |
| Moderate/Severe (WAZ < -2 SDs) | 75 (12.5) | 534 (11.9) |  | 65 (86.7) | 8 (10.7) | 2 (2.7) |  |
| ^a^ The majority of controls selected for CRP testing had an increased likelihood for elevated CRP (i.e., controls that were positive for *S. pneumoniae* on whole blood PCR, had a respiratory tract illness, or had a total nasopharyngeal/oropharyngeal pathogen load in the top 25% of controls at a site were oversampled for testing).  ^b^ Chi-square univariate test. | | | | | | | |
| ^c^ P-value obtained from multivariate logistic regression adjusting for site and age, comparing CRP<40 vs. CRP≥40 mg/l. | | | | | | | |
| ^d^ Respiratory tract illness (RTI) in controls was defined as having cough or runny nose. RTI was also defined as having 1) at least one of ear discharge, wheezing, or difficulty breathing *and* 2) either a measured fever (temp ≥ 38.0°C) within the previous 48 hours, or a history of sore throat. | | | | | | | |
| ^e^ Normal defined as WHO weight-for-age z-score ≥ -2 SDs. Moderate/severe defined as WHO weight-for-age z-score < -2 SDs. | | | | | | | |

| **Supplementary Table 3. Characteristics of HIV-negative, CXR-positive^a^ cases with very high levels of C-reactive protein (CRP)** | | | | | |
| --- | --- | --- | --- | --- | --- |
|  | **CRP <40 mg/L** | **CRP 40-<100 mg/L** | **CRP 100-<200 mg/L** | **CRP ≥200 mg/L** | **P-value**^b^ |
|  | (N=982) | (N=293) | (N=137) | (N=96) |  |
|  | n (col%) | n (col%) | n (col%) | n (col%) |  |
| **Site** |  |  |  |  |  |
| Kenya | 139 (14.2) | 43 (14.7) | 15 (10.9) | 20 (20.8) |  |
| The Gambia | 90 (9.2) | 46 (15.7) | 22 (16.1) | 18 (18.8) |  |
| Mali | 118 (12.0) | 37 (12.6) | 33 (24.1) | 19 (19.8) |  |
| Zambia | 97 (9.9) | 51 (17.4) | 28 (20.4) | 12 (12.5) | .14 |
| South Africa | 293 (29.8) | 81 (27.6) | 27 (19.7) | 26 (27.1) |  |
| Thailand | 69 (7.0) | 16 (5.5) | 6 (4.4) | 0 (0.0) |  |
| Bangladesh | 176 (17.9) | 19 (6.5) | 6 (4.4) | 1 (1.0) |  |
| **Age** (months)  0-5  6-11  11-23  24-59 |  |  |  |  |  |
|  | 422 (43.0) | 106 (36.2) | 41 (29.9) | 21 (21.9) |  |
|  | 233 (23.7) | 79 (27.0) | 28 (20.4) | 19 (19.8) |  |
|  | 223 (22.7) | 71 (24.2) | 48 (35.0) | 32 (33.3) | .002 |
|  | 104 (10.6) | 37 (12.6) | 20 (14.6) | 24 (25.0) |  |
| **Female** | 424 (43.2) | 117 (39.9) | 66 (48.2) | 46 (47.9) | .26 |
| **Very severe pneumonia** | 253 (25.8) | 89 (30.4) | 53 (38.7) | 40 (41.7) | .02 |
| **Malnutrition**^c^ | 338 (34.6) | 100 (34.1) | 68 (49.6) | 34 (35.8) | .02 |
| **Fever^d^** | 755 (77.0) | 268 (91.5) | 124 (90.5) | 93 (96.9) | .15 |
| **Hypoxemia**^e^ | 426 (43.5) | 137 (46.8) | 64 (46.7) | 41 (42.7) | .74 |
| **Died within 30 days**^f^ | 58 (6.5) | 16 (6.3) | 10 (8.6) | 11 (13.4) | .10 |
| **Wheezing** | 352 (35.8) | 62 (21.2) | 16 (11.7) | 10 (10.4) | .37 |
| **Discharge diagnosis** |  |  |  |  |  |
| Pneumonia only | 522 (53.2) | 156 (53.2) | 70 (51.1) | 52 (54.2) | .13 |
| Bronchiolitis | 126 (12.8) | 33 (11.3) | 7 (5.1) | 2 (2.1) | .04 |
| Diarrhea | 13 (1.3) | 9 (3.1) | 3 (2.2) | 0 (0.0) | .76 |
| Malaria | 3 (0.3) | 1 (0.3) | 1 (0.7) | 2 (2.1) | .58 |
| Meningitis | 5 (0.5) | 1 (0.3) | 1 (0.7) | 2 (2.1) | .38 |
| Septicemia | 19 (1.9) | 2 (0.7) | 7 (5.1) | 2 (2.1) | .03 |
| Tuberculosis | 24 (2.4) | 10 (3.4) | 6 (4.4) | 2 (2.1) | .36 |
| Other | 284 (28.9) | 86 (29.4) | 43 (31.4) | 35 (36.5) | .74 |
| ^a^ CXR-positive: evidence of alveolar consolidation, other infiltrate, or both on chest radiograph.  ^b^ Multinomial logistic regression with 3-level outcome of CRP 40-<100 mg/l, 100-<200 mg/l, and ≥200 mg/l; p-value adjusted for age and site. | | | | | |
| ^c^ Moderate or severe malnutrition (WHO weight-for-age z score < -2 SDs). | | | | | |
| ^d^ Observed temperature ≥ 38.0⁰C or fever reported by guardian. | | | | | |
| ^e^ Hypoxemia: room air reading of O_2_ sat <90% at sites at elevation (Zambia and South Africa) and <92% at all other sites or O_2_ requirement (if on O_2_ and room air reading not available). | | | | | |
| ^f^ Died within 30 days of enrollment among cases with known vital status at 30 days. | | | | | |

**Supplementary Table 4. Distribution of C-Reactive Protein (CRP) among HIV-negative PERCH Cases with Various Case Definitions for Bacterial and Viral Etiology**

| **CRP (mg/L)** | **Confirmed bacterial pneumonia cases^a^** | | | | | **RSV pneumonia cases^b^** | | | | |
| --- | --- | --- | --- | --- | --- | --- | --- | --- | --- | --- |
|  | **(increasing specificity for bacterial pneumonia)** | | | | | **(decreasing specificity for RSV pneumonia)** | | | | |
|  | **CXR-Normal** | **All** | **CXR-OI^c^** | **CXR+^d^** | **CXR-AC^e^** | **CXR-Normal** | **All** | **CXR-OI^b^** | **CXR+^c^** | **CXR-AC^d^** |
|  | **N=28** | **N=119** | **N=16** | **N=64** | **N=48** | **N=218** | **N=556** | **N=143** | **N=259** | **N=116** |
|  | n (%) | n (%) | n (%) | n (%) | n (%) | n (%) | n (%) | n (%) | n (%) | n (%) |
| <40 | 7 (25.0) | 28 (23.5) | 8 (50.0) | 15 (23.4) | 7 (14.6) | 193 (88.5) | 459 (82.6) | 118 (82.5) | 198 (76.4) | 80 (69.0) |
| ≥40 | 21 (75.0) | 91 (76.5) | 8 (50.0) | 49 (76.6) | 41 (85.4) | 25 (11.5)^†^ | 97 (17.4)^†^ | 25 (17.5)* | 61 (23.6)^†^ | 36 (31.0)^†^ |
| <10 | 4 (14.3) | 12 (10.1) | 5 (31.3) | 6 (9.4) | 1 (2.1) | 139 (63.8) | 308 (55.4) | 72 (50.3) | 120 (46.3) | 48 (41.4) |
| 10-<40 | 3 (10.7) | 16 (13.4) | 3 (18.8) | 9 (14.1) | 6 (12.5) | 54 (24.8) | 151 (27.2) | 46 (32.2) | 78 (30.1) | 32 (27.6) |
| 40-<100 | 5 (17.9) | 21 (17.6) | 4 (25.0) | 11 (17.2) | 7 (14.6) | 23 (10.6) | 76 (13.7) | 19 (13.3) | 45 (17.4) | 26 (22.4) |
| ≥100 | 16 (57.1) | 70 (58.8) | 4 (25.0) | 38 (59.4) | 34 (70.8) | 2 (0.9) | 21 (3.8) | 6 (4.2) | 16 (6.2) | 10 (8.6) |
| ^a^ Any bacterial pathogen found on blood culture, lung aspirate culture or PCR, pleural fluid culture or PCR, or *S. pneumoniae* detected in pleural fluid by BinaxNow assay. | | | | | | | | | | |
| ^b^ Cases positive for RSV on nasopharyngeal/oropharyngeal (NP/OP) PCR or induced sputum PCR, excluding 1) confirmed bacterial cases (defined above) and 2) cases with whole blood pneumococcal PCR density > 10^2.2^ copies/ml or NP/OP *S. pneumoniae* PCR density > 10^6.9^ copies/ml or NP/OP PCR density of *H. influenzae* > 10^5.9^ copies/ml). | | | | | | | | | | |
| ^c^ CXR-OI: other infiltrate (without alveolar consolidation) identified on chest radiograph. | | | | | | | | | | |
| ^d^ CXR+: alveolar consolidation and/or other infiltrate identified on chest radiograph. | | | | | | | | | | |
| ^e^ CXR-AC: alveolar consolidation identified on chest radiograph, with or without other infiltrates. | | | | | | | | | | |
| *p<.05; ^†^p<.001 (comparing CRP <40 vs. ≥ 40 mg/L within same case sub-group among confirmed bacterial cases vs. RSV+ cases). | | | | | | | | | | |

| **Supplementary Table 5: Distribution of C-reactive protein (CRP) among HIV-negative confirmed *S. pneumoniae*, confirmed *H. influenzae*, and confirmed other bacterial cases.** | | | | | | | |
| --- | --- | --- | --- | --- | --- | --- | --- |
| **CRP (mg/L)** | **Confirmed *S. pneumoniae*^a^** | | **Confirmed *H. Influenzae*^b^** | | **Confirmed Other bacteria^c^** | |  |
|  | Neg^d^ | Pos. | Neg^d^ | Pos. | Neg^d^ | Pos. |  |
|  | n=3232 | n=38 | n=3232 | n=20 | n=3232 | n=62 |  |
| <40 | 2399 (74.2) | 6 (15.8) | 2399 (74.2) | 3 (15.0) | 2399 (74.2) | 19 (30.6) |  |
| ≥40 | 833 (25.8) | 32 (84.2) | 833 (25.8) | 17 (85.0) | 833 (25.8) | 43 (69.4)** |  |
| p-value* | <.001 | | <.001 | | <.001 | |  |
| <10 | 1406 (43.5) | 3 (7.9) | 1406 (43.5) | 1 (5.0) | 1406 (43.5) | 8 (12.9) |  |
| 10-<40 | 993 (30.7) | 3 (7.9) | 993 (30.7) | 2 (10.0) | 993 (30.7) | 11 (17.7) |  |
| 40-<100 | 531 (16.4) | 3 (7.9) | 531 (16.4) | 3 (15.0) | 531 (16.4) | 15 (24.2) |  |
| ≥100 | 302 (9.3) | 29 (76.3) | 302 (9.3) | 14 (70.0) | 302 (9.3) | 28 (45.2)*** |  |
| ^a^ *S. pneumoniae* identified by blood culture, lung aspirate PCR or culture or pleural fluid PCR, culture or BinaxNow. | | | | | | | |
| ^b^ Confirmed *H. influenzae* infection identified by blood culture, pleural fluid PCR or culture, or lung aspirate PCR or culture.  ^c^ Other bacterial pathogen, excluding  *S. pneumoniae* and  *H. influenzae,* identified by blood culture, pleural fluid PCR or culture, or lung aspirate PCR or culture. | | | | | | | |
| ^d^ Excludes all confirmed bacterial cases (n=145).  * p-values from logistic regression for elevated CRP (≥40 vs. <40 mg/L) adjusted for site and age.  ** p-value=.21 comparing percent of cases confirmed for Spn or Hib with CRP ≥40 copies/mL to cases confirmed for other pathogens, adjusted for site and age.  *** p-value=.03 comparing percent of cases confirmed for Spn or Hib with CRP ≥100 copies/mL to cases confirmed for other pathogens, adjusted for site and age.     \|  \| \| --- \| | | | | | | | |

| **Supplementary Table 6. Distribution of C-reactive protein among severe and very severe pneumonia cases with and without suspected *S. pneumoniae* and *H. influenzae* pneumonia^a^** | | | | | | | | |
| --- | --- | --- | --- | --- | --- | --- | --- | --- |
| **CRP (mg/L)** | ***S. pneumoniae* (Spn)** | | | | ***H. influenzae* (HINF)** | | **Spn or HINF** | |
|  | **NP/OP PCR density** | | **Whole blood PCR density** | | **NP/OP PCR density** | | **PCR Density** | |
|  | Low/Neg^b^ | High^b^ | Low/Neg^c^ | High^c^ | Low/Neg^d^ | High^d^ | Low/Neg^e^ | High^e^ |
|  | n=2046 | n=260 | n=2160 | n=94 | n=1693 | n=612 | n=1463 | n=781 |
| <40 | 1542 (75.4) | 144 (55.4) | 1597 (73.9) | 46 (48.9) | 1297 (76.6) | 390 (63.7) | 1143 (78.1) | 501 (64.1) |
| ≥40 | 504 (24.6) | 116 (44.6) | 563 (26.1) | 48 (51.1) | 396 (23.4) | 222 (36.3) | 320 (21.9) | 280 (35.9) |
| p-value^f^ | <.001 | | <.001 | | <.001 | | <.001 | |
| <10 | 897 (43.8) | 83 (31.9) | 926 (42.9) | 25 (26.6) | 791 (46.7) | 189 (30.9) | 699 (47.8) | 254 (32.5) |
| 10-<40 | 645 (31.5) | 61 (23.5) | 671 (31.1) | 21 (22.3) | 506 (29.9) | 201 (32.8) | 444 (30.3) | 247 (31.6) |
| 40-<100 | 318 (15.5) | 52 (20.0) | 353 (16.3) | 13 (13.8) | 245 (14.5) | 124 (20.3) | 208 (14.2) | 149 (19.1) |
| ≥100 | 186 (9.1) | 64 (24.6) | 210 (9.7) | 35 (37.2) | 151 (8.9) | 98 (16.0) | 112 (7.7) | 131 (16.8) |
|  | | | | | | | | |
| ^a^Suspected bacterial pneumonia cases were children with NP/OP PCR *Streptococcus pneumoniae* (Spn) density >10^6.9^ copies/ml, whole blood PCR Spn density >10^2.2^ copies/ml, or NP/OP PCR *Haemophilus influenzae* (Hinf) density >10^5.9^ copies/ml. Table excludes all confirmed bacterial cases (n=119), all confirmed viral cases (n=9), and all cases positive for RSV by NP/OP PCR or induced sputum PCR (n=883). | | | | | | | | |
| ^b^ Low density/Negative Spn: NP/OP PCR density ≤ 10^6.9^ copies/ml; high density Spn: NP/OP PCR density > 10^6.9^ copies/ml.  ^c^ Low density/Negative Spn: whole blood PCR density ≤ 10^2.2^ copies/ml; high density Spn: whole blood PCR density > 10^2.2^ copies/ml.  ^d^ Low density/Negative HINF: NP/OP PCR density ≤ 10^5.9^ copies/ml; high density Spn: NP/OP PCR density > 10^5.9^ copies/ml.  ^e^ Low density/Negative: Spn NP/OP PCR density ≤ 10^6.9^ copies/ml *and* Spn whole blood PCR density ≤ 10^2.2^ copies/ml *and* HINF NP/OP PCR density ≤ 10^5.9^ copies/ml; high density: Spn NP/OP PCR density > 10^6.9^ copies/ml *or* Spn whole blood PCR density > 10^2.2^ copies/ml *or* HINF NP/OP PCR density > 10^5.9^ copies/ml.  ^f^ Multivariate logistic regression adjusted for site, age. | | | | | | | | |
| Compared to cases without high density for Spn or Hinf, the proportion with CRP ≥40 mg/L was higher among suspected bacterial cases (36% vs. 22%, p<.001). Of the three density measures, the association with elevated CRP was strongest for high density Spn in whole blood: 51% and 37% of cases had CRP ≥40 and ≥100 mg/L, respectively, compared to 26% (p<.001) and 10% (p<.001) of cases who had low density Spn in whole blood or were negative). | | | | | | | | |

- **Acknowledgements:**
- ***Pneumonia Methods Working Group.*** Robert E Black, Zulfiqar A Bhutta, Harry Campbell, Thomas Cherian, Derrick W Crook, Menno D de Jong, Scott F Dowell, Stephen M Graham, Keith P Klugman, Claudio F Lanata, Shabir A Madhi, Paul Martin, James P Nataro, Franco M Piazza, Shamim A Qazi, and Heather J Zar.
- ***PERCH Expert Group.*** William C. Blackwelder, Harry Campbell, John A. Crump, Adegoke Falade, Menno D. de Jong, Claudio Lanata, Kim Mulholland, Shamim Qazi, Cynthia G. Whitney.
- ***PERCH Chest Radiograph Reading Panel***
- **Readers:** Dr. Kamrun Nahar, Dr. Fariha Bushra Matin, Dr. Claire Oluwalana, Dr. Bernard Ebruke, Dr. Joyce Sande, Dr. Micah Silaba Ominde, Dr. Mahamadou Diallo, Dr. Breanna Barger-Kamate, Dr. Nasreen Mahomed, Dr. David P. Moore, Dr. Anchalee Kruatrachue, Dr. Piyarat Suntarattiwong, Dr. Musaku Mwenechanya, Dr. Rasa Izadnegahdar, **Arbitrators:** Dr. Vera Manduku, Dr. John DeCampo, Dr. Marg DeCampo, Dr. Fergus Gleeson.
- ***PERCH Contributors:***
- **Bangladesh:** Kamrun Nahar, Arif Uddin Sikdir, Sharifa Yeasmin, Dilruba Ahmed, Muhammad Ziaur Rahman, Muhammad Yunus, Muhammad Al Fazl Khan, Muhammad Jubayer Chisti, Abu Sadat Muhammad Sayeem, Shahriar Bin Elahi, Mustafizur Rahman; **The Gambia:** Michel Dione, Emmanuel Olutunde, Peter Githua, Ogochukwu Ofordile, Rasheed Salaudeen, David Parker; **Kenya:** Shebe Mohamed, Siti Ndaa, Micah Silaba, Neema Muturi, Angela Karani, Sammy Nyongesa, Anne Bett, Daisy Mugo, Salim Mwarumba, Robert Musyimi, Andrew Brent, James Nokes, David Mulewa, Joyce Sande, John Odhiambo, Joshua Wambua, Nuru Kibirige, Caroline Mulunda, Hellen Mjalla, Norbert Katira, Karen Dama, Loice Masha, Christine Mutunga, Mwanajuma Ngama, Stephen Mangi, Riziki Anthony, Mwarua Yubu, Elijah Wakili, Benson Katana, Shoboi Mgunya, Emmanuel Mumba, Benedict Mver, George Kuria, Felix Githinji, Norbert Kihuha, Boniface Jibendi, Tahreni Bwanaali, Agustus Kea; **Mali:** Nana Kourouma, Aliou Toure, Mahamadou Diallo, Breana Barger-Kamate, Mariam Samake, Seydou Sissoko, Abdoul Aziz Maiga, Mariam Samake, Toumani Sidibe, Mariam Sylla, Aziz Diakite, Bassirou Diarra; **South Africa:** Azwidihwi Takalani, Andrea Hugo, Susan Nzenze, Ndulela Titi, Mmabatho Selela, Malebo Motiane, Minah Nkuna,Nonhlanhla Tsholetsane, Sibonsile Moya, Debra Katisi, Tondani Netshishivhe, Lerato Mapetla, Gudani Singo, Simphiwe Gasa, Cece Mgenge, Nozipho Mthunzi, Nombulelo Monedi, Tanja Adams, Shafeeka Mangera, Jeannette Wadula, Peter Tsaagane, Jenifer L. Vaughan, Sakina Loonat, Martin Hale, Sugeshnee Pather, Mariëtte Middel, Siobhan Trenor, Palesa Morailane, Ntombi Maya, Rene Sterley, Charné Combrinck, Given Malete, Lerato Qoza, Grizelda Liebenberg, Hendrik van Jaarsveld, Zunaid Kraft, Lisa-Marie Mollentze, Lourens Combrinck, Tsholofelo Mosome; **Thailand:** Sununta Henchaichon, Dr. Tussanee Amornintapichet, Dr. Somchai Chuananont, Toni Whistler, Juraiporn Ratanodom, Patranuch Sapchookul, Ornuma Sangwichian, Sirirat Makprasert, Manoon Hirunsalee, Possawat Jorakate, Anek Kaewpan, Duangkamol Siludjai, Apiwat Lapamnouysup, Dr. Wantana Paveenkittiporn, Waraporn Ubonphen, Dr. Peera Areerat, Ms.Yupapan Wannachaiwong, Ms Tewa Faipet, Ms Punnat Natnarakorn, Ms Ahchanan Sacharone, Mr.Winai Makmool, Ms. Kanlaya Sornwong, Ms. Promporn Sansuriwong, Ms. Ratchanida Potiya, Ms. Wasana Hongsawong, Ms.Wipa Matchaikhen, Ms. Thatsanawan Chaiyabil, Ms.Piyapai Wannarach, Ms Chamaiporn Wadeesirisak, Mr. Yuttapong Norapet, Mattana Bangkung, Mr. Barameht Piralam, Sathapana Naorat, Anchalee Jatapai, Prasong Srisaengchai, Dr. Leonard Peruski, Ms.Dawan Phaensoongnoen, Ms.Tussaaorn Klangprapan, Ms.Narawadee Dumrongdee, Ms.Atchara Srithongkham, Mr. Piyawut Noinont, Ms. Pornthip Kamlee, Ms.Siyapa Mongkornsuk; **Zambia:** Justin Mulindwa, Musaku Mwenechanya, John Mwaba, Magdalene Mwale, Julie Duncan, Kazungu Siazele, Muntanga Mapeni, Emily Hammond; **Canterbury Health Laboratory, Christchurch, New Zealand:** Rose Watt, Shalika Jayawardena.
